# Origination of an X-Linked Testes Chimeric Gene by Illegitimate Recombination in *Drosophila*

**DOI:** 10.1371/journal.pgen.0020077

**Published:** 2006-05-19

**Authors:** J. Roman Arguello, Ying Chen, Shuang Yang, Wen Wang, Manyuan Long

**Affiliations:** 1 Committee on Evolutionary Biology, University of Chicago, Chicago, Illinois, United States of America; 2 Department of Ecology and Evolution, University of Chicago, Chicago, Illinois, United States of America; 3 Chinese Academy of Sciences–Max Planck Junior Scientist Group, Key Laboratory of Cellular and Molecular Evolution, Kunming Institute of Zoology, Kunming, Yunnan, China; Stowers Institute for Medical Research, United States of America

## Abstract

The formation of chimeric gene structures provides important routes by which novel proteins and functions are introduced into genomes. Signatures of these events have been identified in organisms from wide phylogenic distributions. However, the ability to characterize the early phases of these evolutionary processes has been difficult due to the ancient age of the genes or to the limitations of strictly computational approaches. While examples involving retrotransposition exist, our understanding of chimeric genes originating via illegitimate recombination is limited to speculations based on ancient genes or transfection experiments. Here we report a case of a young chimeric gene that has originated by illegitimate recombination in *Drosophila.* This gene was created within the last 2–3 million years, prior to the speciation of *Drosophila simulans, Drosophila sechellia,* and *Drosophila mauritiana.* The duplication, which involved the *Bällchen* gene on Chromosome 3R, was partial, removing substantial 3′ coding sequence. Subsequent to the duplication onto the X chromosome, intergenic sequence was recruited into the protein-coding region creating a chimeric peptide with ~ 33 new amino acid residues. In addition, a novel intron-containing 5′ UTR and novel 3′ UTR evolved. We further found that this new X-linked gene has evolved testes-specific expression. Following speciation of the D. simulans complex, this novel gene evolved lineage-specifically with evidence for positive selection acting along the D. simulans branch.

## Synopsis

Illegitimate recombination, the non-homologous recombination that occurs between DNA sequences with few or no identical nucleotides, is a general phenomenon that has been known to cause many medically important deleterious changes. However, little is known about the positive side of such a process. For example, little is known about its relative role in the origin of new gene functions that confer increased fitness to species. This work contributes to the understanding of the significance of this process. Here the authors report on a young chimeric gene that has originated by illegitimate recombination in *Drosophila.* The term “chimeric gene” refers to gene structures—both coding and noncoding—which have been generated from distinct parental loci. This chimeric gene was created within the last 2–3 million years, prior to the speciation of *Drosophila simulans, Drosophila sechellia,* and Drosophila mauritiana. A gene on Chromosome 3R was duplicated onto the X chromosome and recruited intergenic sequence, creating a chimeric peptide. It was found that this new X-linked gene has evolved testes-specific expression. Following speciation of the D. simulans complex, this novel gene evolved lineage-specifically under positive Darwinian selection.

## Introduction

The study of the origin of novel genes is crucial to understanding the evolution of biological diversity. Two general evolutionary routes responsible for creating new genes have been postulated. Historically, gene duplication was the first mechanism to be considered in the 1930s [1–3], followed by a general model of the process by Ohno [4]. Ohno's classical model states that while one copy maintains the ancestral function, the other copy can accumulate new mutations that may eventually lead to the origin of new functions [4]. The second route is through the formation of chimeric genes. Chimeric genes can be formed by exon or domain shuffling [5,6], in which recombination among different domains and exons can generate new genes structures [6–10]. They can also be formed through the combination of genic and nongenic sequences, as well as through scenarios that include both shuffling events and nongenic incorporations [11].

The first genetic mechanism proposed for exon shuffling was illegitimate recombination [5]. Illegitimate (nonhomologous) recombination refers to an array of genetic mechanisms that are united by their ability to integrate genomic DNA (gDNA) while relying on little or no sequence homology. Regardless of the particular steps involved, chimeric genes formed by illegitimate recombination are formed on the gDNA level. For the precise definition of exon shuffling to apply, recombination within introns is required [5]. While many cases of exon shuffling have been identified [8,9], chimeric gene formation also occurs through the shuffling of domains, which may or may not be generated through intron-facilitated recombination [12]. In addition, it has also been recognized that chimeric genes can be created through retrotransposition and thus through an intermediate RNA step [11,13,14].

Transfection experiments, along with experimentally produced chimeric genes, using bacteria, yeast, and mammalian systems, have revealed likely molecular mechanisms involved in illegitimate recombination events resulting in chimeric gene structures [15–20]. Also adding to our mechanistic insight are well-documented instances of illegitimate recombination events giving rise to disease-related genes [21–23]. However, the antiquity of the identified non-deleterious chimeric genes precludes investigation into the evolutionary forces that operated on them during their early phases and that have led to their fixations. It has become clear that in order to probe this critical period of a chimeric gene's history newly evolved examples are requisite.

Chimeric genes can create a wide range of new functions [9,13,24,25]. Recently, many cases of male-specific functions recently evolved by retroposition in mammals and fruit flies have been documented [26–29]. These genes have evolved testis expression that must have resulted from the new regulatory sequences required by chimeric retrogenes. Evolutionary analyses have provided evidence that natural selection for sex-related functions, and not mutational biases, is the driving force for their male-biased expression [26,28]. This suggests that natural selection for new sex-related functions might be independent of the molecular processes that create chimeric genes.

Here we report the first case in *Drosophila* that demonstrates how a recent illegitimate recombination event has created a chimeric gene that has evolved a new sex-specific function. We have chosen to name this gene *Hun Hunaphu (Hun)* after a fertility god from Mayan mythology. Hun Hunaphu possessed such fertility, that after being severed, his head was placed on a dead gourd and induced the production of healthy fruit. Our analyses demonstrate that *Hun* originated either prior to or during the speciation of *Drosophila simulans, Drosophila sechellia,* and Drosophila mauritiana. Its coding region is composed of a partial duplication of *Bällchen* (*CG6386,* Chromosome 3R), which has been shown to encode a kinase that is involved in germ cell development (FlyBase; http://flybase.bio.indiana.edu/), and recruited intergenic X chromosome sequence. The newly evolved 3′ and 5′ UTRs were also found to have originated from intergenic X sequence. Expression studies revealed that along with these structural changes, *Hun* evolved testes-specific expression. Since speciation, *Hun* has evolved lineage-specifically: the *D. simulans'* copy has evolved a new protein-coding gene while the D. mauritiana and D. sechellia copies may have degenerated into pseudogenes by accumulating deletions and multiple premature stop codons. There is evidence from evolutionary analyses that the D. simulans protein-coding copy evolved under positive selection. Interestingly, our data for *Hun* in D. simulans are consistent with Rice's sexual antagonism hypothesis [30], in which a mutation that is selectively advantageous in one sex, while being deleterious in the other, will be more likely fixed on the X chromosome.

## Results

### *Hun* Is Present in *D. sechellia, D. mauritiana,* and D. simulans

**Figure 1:**
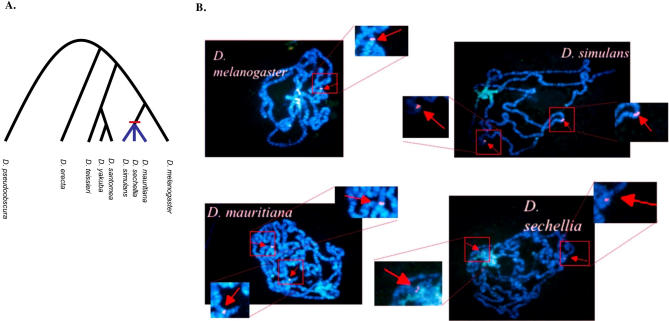
D. melanogaster Subgroup Phylogeny with the *Hun* Duplication Mapped onto It as Informed by FISH (A) D. melanogaster phylogeny with Drosophila pseudoobscura as the out-group. Red bar indicates *Hun* duplication, with blue branches below it noting the species in possession of it. (B) FISH was carried out on the D. melanogaster subgroup. Probe signals are indicated by the red arrows. The two signals found in *D. sechellia, D. mauritiana,* and D. simulans indicate the *Hun* duplication. Only one signal is found in *D. melanogaster,* the out-group in this figure, as well as for all other species in the subgroup. FISH images for full subgroup will be provided upon request.

**Figure 2:**
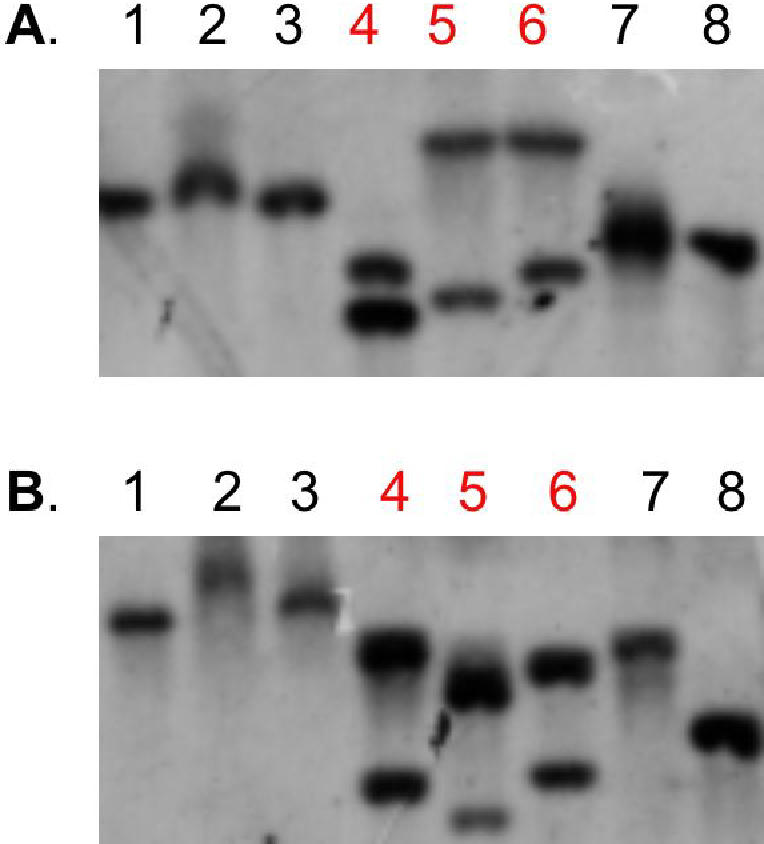
Southern Hybridization Verifying the *Hun* Duplication in *D. sechellia, D. simulans,* and D. mauritiana Hybridizations, using genomic DNA from the D. melanogaster subgroup and the FISH cDNA probes, are contained in lanes 1–8: (1) *D. teissieri,* (2) *D. santomea,* (3) *D. yakuba,* (4) *D. simulans,* (5) *D. sechellia,* (6) *D. mauritiana,* (7) *D. erecta,* and (8) D. melanogaster. Species for which two signals were recovered *(D. mauritiana, D. sechellia,* and *D. simulans)* are noted with red numbers. Hybridization A (top) was carried out using the BamH I restriction enzyme; hybridization B (bottom) was carried out using the Xho I restriction enzyme. These results are in agreement with the FISH results (see Figure 1).

The identification and verification of the duplication proceeded in four steps: fluorescent in situ hybridization (FISH), Southern analysis, BLAST searches, and genomic amplification and sequencing. By applying these methods to the Drosophila melanogaster subgroup (Figure 1A), an estimate of the time of the duplication was also achieved from the phylogenetic distribution of the new gene [31,32]. The results of the FISH experiments for *D. melanogaster, D. simulans, D. sechellia,* and D. mauritiana are shown in Figure 1B (FISH results for the full subgroup are available upon request). The cDNA probe for *Bällchen* produced an extra hybridization signal in the D. simulans complex, while only a single signal was found outside of it. The Southern analyses using both BamH I and Xho I restriction enzymes yielded results consistent with the FISH results: two bands were observed only in *D. sechellia, D. mauritiana,* and D. simulans (Figure 2A and 2B). The tBLASTN searches using the available genome sequences of *D. melanogaster, D. simulans,* and Drosophila yakuba also supported each of our previous findings and indicated that the direction of the duplication was to the X chromosome. An alignment using the Vista Browser [33–35] of the X chromosome between D. simulans and D. melanogaster places *Hun* between the predicted genes *CG32614* and *CG12454.* Finally, our gene-specific primers were successful in amplifying *Hun* from each of the three species.

### *Hun* Has Recruited Intergenic X Chromosome Sequence into Its Coding Region and into Its 3′ and 5′ UTR Region

The duplication event onto the X chromosome shortened the 1,867 base pair (bp) *Bällchen* gene by ~ 412 bp, and included only ~ 65 bp 5′ of the original start codon (Figure S1). In total, it was a duplication of ~ 1,520 bp. Inspection of the available D. simulans genomic sequence made clear that *Bällchen*'s stop codon had not been included in the duplication and an open reading frame continued for ~ 99 bp (33 amino acid residues) into the flanking X chromosome. This led us to suspect that a novel 3′ coding region had been recruited. Based on this information, we designed primers downstream of the first putative stop codon. Through both manual and computational approaches (REPuter [36]), no 3′ polyA tract or direct repeats were found in the duplication's flanking regions. Because the survival of a partial duplication requires the evolution of new regulatory regions, we investigated the possibility that *Hun* recruited flanking X chromosome sequence into its UTRs. Using RNA from *D. sechellia, D. mauritiana,* and *D. simulans,* we carried out 3′ RACE and 5′ RLM-RACE experiments. For all three species we obtained 3′ reads that extended to an identifiable polyadenylation site (Figure S1). The amount of intergenic X chromosome sequence that was recruited into the coding region was verified to be ~ 99 bps long with an additional ~ 167 bps to the polyadenylation site.

From the same three species, we were able to successfully carry out the 5′ RLM-RACE only on D. simulans. Using an annealing temperature gradient from 50 °C–70 °C for the first round of PCR, we produced six distinct bands, all of which were purified and sequenced. Of the six bands, four were clearly overlapping fragments and possessed the RACE Outer primer as well as our gene-specific primer. The total length of these reads ranged from 143–404 bp long. Of the remaining two products, one was a nonspecific and the other failed in the sequencing reaction. By aligning the longest of the four products (or the consensus sequence for the four products) to D. simulans gDNA, an intron of 49 bps with a standard splicing signal at the two ends (GT/AG) was identified (Figure S2). It is notable that NNPP [37] identified seven putative promoters, and that the second highest scoring prediction has a transcription start site that disagrees with our 5′ RLM RACE results by only a single base. This provides additional evidence that our 5′ RLM RACE experiments did extend out to the new transcription start sites. Finally, no evidence for a signal peptide was found using our ten *D. simulans Hun* peptides (unpublished data).

We remained curious about the origin of the UTR regions. In particular, the presence of the 5′ UTR containing an intron led us to wonder if there was an existing unidentified gene or gene fragment in the region between *CG32614* and *CG12454,* or if there was evidence that the recruited regions shared homology to other expressed sequences. To investigate this, we queried the GenBank's EST database [38] using our 5′ UTR as well as the *Hun* locus with the *Bällchen* region removed. Neither of these queries returned significant matches (unpublished data).

### *Hun* Has Evolved Lineage-Specifically

Our efforts to amplify and sequence *Hun* from the three species revealed a significant size difference between D. sechellia and the other two species. An alignment of the full *Hun* gene sequences from each of the three species revealed that the gene structure has evolved differently (Figure S3). D. simulans maintains a single open reading frame, while both D. sechellia and D. mauritiana have sustained deletions leading to seven and six premature stop codons, respectively. In *D. sechellia,* this has been caused by three large and one small deletion in the center of the gene. In *D. mauritiana,* the frame shift was caused by a single base deletion. Despite these mutations, the 5′ and 3′ ends, including the newly recruited coding region, are well conserved (see Figures S1 and S2 for details).

In light of these results for the single D. sechellia and *D. mauritiana Hun* samples, we wanted to know if the mutations were fixed. To address this, we carried out screens for the deletions in additional samples. Because we know the size of the deletions in *D. sechellia,* we carried out simple PCR screens for size differences when compared to the homologous *D. simulans Hun* region. We carried this out in six additional D. sechellia lines and in each case observed the deletions. We also carried out a sequencing screen for D. mauritiana's single nucleotide deletion on nine additional lines. The deletion was present in all individuals (image and alignments provided upon request).

### *Hun* Has Evolved Testes-Specific Expression

**Figure 3:**
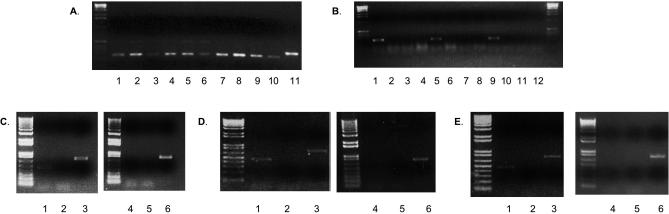
Expression Study Results for the *Bällchen and Hun* gene (A) *Bällchen* is expressed ubiquitously in both *D.melanogaster* and *D. simulans:* whole body, D. melanogaster (1), D. simulans (2); head, D. melanogaster (3), D. simulans (4); thorax, D. melanogaster (5), *D. simulans* (6); testes, D. melanogaster (7), D. simulans (8); and accessory gland with ejaculatory duct, D. melanogaster (9), D. simulans (10). Lane 11 is a genomic control. (B) RT-PCR results for *Hun* from adult male and female *D. simulans, D. mauritiana,* and D. sechellia flies. *Hun*'s expression is limited to *D. simulans, D. mauritiana,* and D. sechellia males, lanes 1, 5, and 9, respectively. Lanes 3, 7, and 11 are (D) *D. simulans, D. mauritiana,* and D. sechellia females. Lanes 2, 4, 6, 8, 10, and 12 are corresponding RT-controls. (C–E) Tissue specific RT-PCR results for the *Hun* gene in D. simulans (C), D. mauritiana (D), and D. sechellia (E). For these RT-PCR experiments testes were dissected from the rest of body. *Hun*'s expression is testes-specific for each species: testes, lane 1; RT-control, lane 2; *Gapdh-2* positive control, lane 3; rest of body, lane 4; RT-control, lane 5; *Gapdh-2* positive control, lane 6.

We investigated the expression profile of *Hun* as well as characterized changes in expression from that of *Bällchen.* Whole-body RT-PCR experiments were carried out over eight species in the D. melanogaster subgroup (Drosophila orena was excluded), separating males and females*.* We began with RT-PCR experiments for *Bällchen* and found it to be expressed in both sexes in all species (image available upon request). To get tissue-specific expression data, we chose as representative species D. melanogaster and D. simulans. We performed dissections, separating tissue into four categories: head, thorax, testes, and accessory gland with ejaculatory duct. Results showed that *Bällchen* is expressed ubiquitously in both species (Figure 3A).

To examine the expression pattern of *Hun,* we followed a similar procedure as above. Males and females were separated, and whole-fly RT-PCR was carried out over the D. melanogaster subgroup. The results demonstrated that *Hun*'s expression in *D. sechellia, D. mauritiana,* and D. simulans is limited to males (Figure 3B). Tissue-specific RT-PCR revealed that the gene's expression is testes-specific for each of the three species (Figure 3C–E).

### Divergence and Polymorphism Analyses

To determine if there is evidence for functional constraint at the DNA sequence level, we inspected the divergence between *Bällchen* and *Hun* within each species using the statistic Ka/Ks. Ka/Ks values for all paralogous comparisons, including D. mauritiana and *D. sechellia,* suggested that *Hun* is conserved. The Ka/Ks value for the comparisons between *Bällchen* and an average *Hun* allele from D. simulans is 0.47, statistically less than one, suggesting functional constraint (Table S1 lists values for all comparisons). When unalignable sequence and premature stop codons are removed from the D. sechellia and D. mauritiana copies, this was also the case (0.57 and 0.15, respectively). Our more sensitive test for constraint, which was based on an expectation from the number of synonymous and nonsynonymous sites found in the *Hun* population dataset and the observed polymorphisms, also supports *Hun* being functionally constrained through higher synonymous nucleotide diversity than nonsynonymous nucleotide diversity (χ^2^ = 8.148, *p* = 0.0058).

To inspect the polymorphism frequency spectrum, we sequenced the entire *Bällchen* and *Hun* gene regions as well as some 3′ and 5′ flanking regions for ten D. simulans lines. Data descriptions and summary statistics are contained in Table 1 (see also Figures S4 and S5). For the parental *Bällchen* sequences, we observed ten haplotypes and low codon bias (0.345). Our results for the Fu and Li's D [39], Fu and Li's F [39], and Tajima's D [40], though negative, were nonsignificant (for each test, *p* > 0.10). We carried out analogous calculations on the ten *Hun* sequences and again obtained ten haplotypes, low codon bias (0.322), and negative but nonsignificant test values (for each test, *p* > 0.10). We then conducted a test of neutrality by contrasting divergence and polymorphism using the McDonald-Kreitman test [41] with polymorphism from the pooled *D. simulans Hun* alleles and the pooled *D. simulans Bällchen* alleles. This test revealed a significant excess of amino acid replacement substitutions (Fisher's exact test *p* = 0.0165, Table 2). However, by pooling the data we are including alleles that are experiencing different effective population sizes (X-linked genes experience a population size 3/4 that of autosomal-linked genes), and thus they may not be experiencing the same levels of purifying selection, mutation, and drift. We therefore also wanted to conduct the McDonald-Kreitman test in the *Hun* lineage only.

**Table 1:**
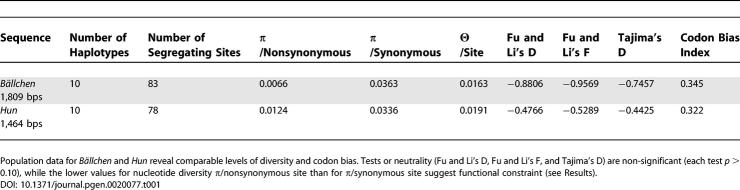
Polymorphism Data for *D. simulans Bällchen* and *D. simulans Hun*

**Table 2:**
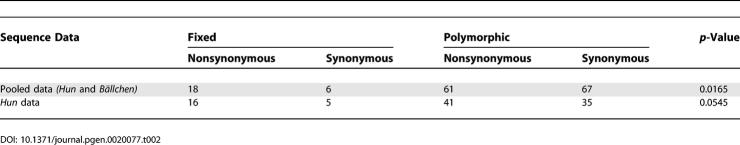
McDonald-Kreitman Tests for Pooled and *Hun*-Only Data

**Figure 4:**
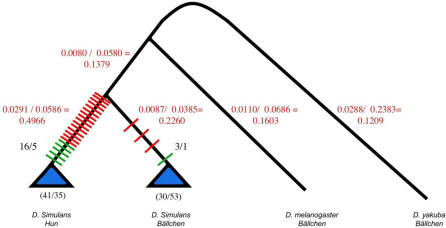
Gene Tree for *D. simulans Hun* and *D. simulans, D. melanogaster,* and D. yakuba's *Bällchen* The tree includes measurements of divergence as measured by Ka/Ks (red ratios), nonsynonymous and synonymous fixations found along the *Hun* and *Bällchen* branches depicted by colored bars (red represents nonsynonymous changes and green represents synonymous changes, black ratio), and polymorphisms found in the D. simulans population data (black ratios below triangles, nonsynonymous/synonymous). The low divergence estimates suggest that all genes are constrained. The most notable feature of the tree is the significant excess of nonsynonymous substitutions along *Hun*'s branch. This excess was detected by McDonald-Kreitman tests, and is significantly different than that of the pooled *Bällchen* and *Hun* data, and marginally significantly different than that of the *Hun*-only data (see Table 2).

To investigate the distribution of the nucleotide substitutions among the paralogous copies, we used the parsimony approach to assign all fixed mutations between the paralogs to their appropriate branches. To achieve this we extracted *Bällchen* from D. yakuba using Wise2 [42], and used it, along with a *D. melanogaster Bällchen* copy, as out-groups to *D. simulans Hun* and *D. simulans Bällchen.* The McDonald-Kreitman test again revealed an excess of amino acid substitutions along the *Hun* branch: fixed replacement substitutions/fixed synonymous substitutions = 16/5 versus polymorphic replacement changes/polymorphic synonymous changes = 41/35 (Fisher's exact test *p* = 0.0545, Figure 4 and Table 2; counting the first base of the coding sequence as 1, these 16 sites are: 130, 298, 531, 685, 797, 877, 931, 973, 1,081, 1,090, 1,123, 1,181, 1,268, 1,277, 1,304, 1,370). This is consistent with the observed elevated replacement substitution rate in the *Hun* branch compared to that of *Bällchen*'s (16 versus 3, respectively, *p* < 0.05). Using the additional *D. yakuba Bällchen* copy, we estimated evolutionary rates for this expanded gene tree and mapped both divergence and population data onto it, providing a detailed picture for the history of these genes (Figure 4).

Inferences of gene conversion between *Hun* and *Bällchen* were made by calculating the number of shared polymorphisms between the two genes from our D. simulans population data, by estimating the rate of gene conversion, *Ĉ*, using the methods of Innan [43], and by analyzing all pair-wise comparisons (ignoring those between the same loci) for conversion tracts using GENECONV [44]. Evidence for conversion events was found in the number of shared polymorphisms and through the estimate of *Ĉ*. We found seven shared polymorphisms; five of these were at synonymous sites and two were at replacement sites (counting the first site of *Hun*'s coding region as 1, these five sites are: 217, 478, 485, 520, 623). *Ĉ* was estimated to be 0.266. No significant tracts were identified through the pair-wise analyses using GENECONV (unpublished data).

## Discussion

Illegitimate recombination has commonly been invoked as an important genetic mechanism driving modular protein evolution throughout the tree of life [5,6,8–10,45]. However, little is known about its contribution to the formation of new genes over a recent evolutionary timescale, or about its relative frequency when compared to alternative mechanisms in leading to new genes. To our knowledge, a detailed example demonstrating how such a process occurs in its early stages has not been reported. Existing examples of module proteins are ancient and thus do not maintain signatures of the evolutionary forces that have acted on them [6], or they remain computational predictions and thus difficult to make functional inference about [45]. Exceptions are instances of well-documented disease genes, which do not contribute to the evolution of new function [22,46] or are limited to transfection experiments [1,18–20,47].

Here we present an identification and evolutionary analysis of a young chimeric gene found in *D. simulans, D. sechellia,* and D. mauritiana that we have named *Hun*. *Hun* has arisen by an illegitimate recombination event from Chromosome 3R to the X, and has incorporated intergenic X chromosome sequence into both its coding region and its UTRs. With these events, *Hun* has evolved testes-specific expression and thus provides a detailed example of a gene evolving new structural and regulatory elements leading to sex-related functions. We discuss the lineage-specific evolution of *Hun* within the D. simulans complex and consider the data in light of gene traffic and the X chromosome.

### *Hun*'s Origin and Chimeric Structure

**Figure 5:**
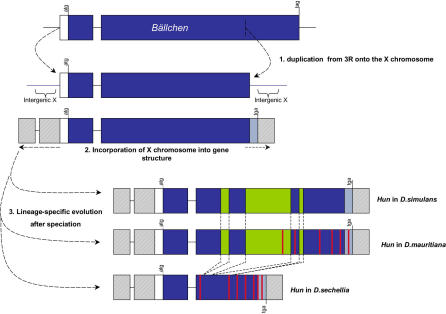
A Model for the Origin and Evolution of *Hun* Striped boxes indicate newly acquired UTR regions, white boxes indicate the *Bällchen* 5′ region included in duplication, grey boxes indicate newly acquired protein-coding regions, green boxes indicate regions deleted from the D. sechellia copy, and red bars represent premature stop codons.

A schematic of *Hun*'s origin and evolution is provided in Figure 5. A duplication event gave rise to ~ 1,520-bp duplication composed of ~ 65 bp 5′ of *Bällchen*'s start codon, but cut short its 1,809-bp coding region by ~ 412 bp. Several lines of evidence indicate that illegitimate recombination led to the formation of *Hun:* presence of *Bällchen*'s only intron, the lack of a 3′ polyA tract, and the absence of direct repeats at *Hun*'s 3′ and 5′ ends. The precise steps involved in illegitimate recombination are varied and are still not fully understood [1,16–20,47]. An explanation for *Hun* may be considered through a model similar to Richardson et al. [48] in which recombination occurs between nonhomologous chromosomes through the non-allelic homologous recombination of low copy repeats (LCRs). A double-strand break occurs in one of the two chromosomes (the X) near the LCR, followed by strand invasion of homologous sequence belonging to the intact chromosome (3R). Strand extension would carry on before rejoining its own chromosome (the X) at either more distal regions of homology or nonhomology. This model accounts for interchromosomal recombination and duplication while avoiding crossovers. The difference is that we do not evoke a role for LCRs. Alternatively, a linear piece of DNA could have been generated as a result of an error in replication involving the *Bällchen* locus on Chromosome 3R. The insertion site on the X chromosome could have been achieved through a double-stranded nick by a topoisomerase. Topo I has been implicated as having a major role in illegitimate recombination, and preferred sites ([g/c][a/t]t) have been identified at 5′ or 3′ insertion sites, and sometimes both [47]. We have identified one putative site at the 5′ insertion site (Figure S1). Finally, the integration of the freed *Bällchen* fragment would have occurred by either the joining of blunt ends or through the pairing of some small number of homologous nucleotides, followed by ligation and filling.

In each of the three species, ~ 99 nucleotides of flanking X chromosome were incorporated into the 3′ -coding region and an additional ~167 bps beyond the stop codon to the polyadenylation site have evolved into a novel 3′ UTR region. 5′ RACE on D. simulans also uncovered a novel 5′ UTR that contains an intron. This is similar to the finding of Begun [49], in which a 5′ UTR from an unknown gene was recruited into *Adh-Finnegan*. The recovery of multiple 5′ RACE products suggests that *Hun* may have multiple transcription initiation sites. However, we were able to identify a putative promoter region that supported the longest RACE product (Figure S2). The intron identified in *Hun*'s 5′ UTR is curious, though the presence and evolution of introns in 5′ UTR regions is not unheard of and have occasionally been shown to play important regulatory roles [50,51]. It is possible that this intron has functional importance, but such a claim remains speculative and further experiments would be needed to demonstrate this. Further, it is not clear if this intron belonged to an ancient module which *Hun* was able to incorporate, or if the structure evolved completely de novo. Our negative results from querying GenBank's EST database, along with the high quality of D. melanogaster genome annotation, lend some weight to it evolving de novo.

Because we were unable to carry out 5′ RACE on the other two species, we cannot be sure that the same structure exists for D. sechellia and *D. mauritiana*. However, it is notable that both the 3′ and 5′ ends of the new coding structure are the most conserved (Figure S3), and that all three copies have evolved the same expression pattern (Figure 3C–E). It seems likely that much of the chimeric structure evolved either prior to or during the early stage of the D. simulans complex speciation.

After the speciation of *D. simulans, D. sechellia,* and *D. mauritiana,* the *Bällchen* portion of *Hun* evolved in a rapid lineage-specific manner (Figures 5 and Figure S3). Most drastically, D. sechellia experienced three significant deletions. These deletions have led to a frame shift and seven premature stop codons. While D. mauritiana has only a single base deletion, it has led to a frame shift and six premature stops codons. Because the *D. simulans'* copy remains an intact open reading frame, the deletions and nonsense mutations have occurred very recently. Even so, our assays for the frame shift mutations in additional populations from both species provide evidence that they are fixed. It is thus a concern whether *Hun* is functional in D. sechellia and D. mauritiana (see below).

### Molecular Evolution and Population Genetics of *Hun*

Molecular evolutionary sequence analyses between *Bällchen* and *Hun* suggest that *Hun* is under functional constraint in all three species (Figure 4). The Ka/Ks ratio between *Bällchen* and *Hun* in D. simulans is below the conservative cutoff of 0.5. This analysis of sequence divergence, revealing the functional constraint in the *D. simulans Hun,* is further supported by the distribution of polymorphisms, which reveals higher synonymous nucleotide diversity than nonsynonymous nucleotide diversity (χ^2^ = 8.148, *p* = 0.0058). Interestingly, the Ka/Ks ratios in the D. sechellia and *D. mauritiana Hun* genes show evidence of constraint despite evidence (multiple premature stop codons) that they may be on their way to becoming pseudogenes (D. sechellia comparison = 0.577; D. mauritiana comparison = 0.13). Two scenarios are likely: This may reflect a constraint that persisted until the genes were degenerated into pseudogenes recently by nonsense mutations. The alternative possibility is that the genes are functional with the nonsense mutations spliced out, as has been found previously [32].

Our population survey using ten Madagascar D. simulans lines uncovered ten *Bällchen* haplotypes (Table 1). Ten haplotypes were also found in our sample for *Hun*. While polymorphism data alone provided no evidence for directional selection in the recent history of either *Bällchen* or *Hun* (Table 1), the McDonald-Kreitman tests provide significant results, revealing an excess of nonsynonymous substitutions. When mapped onto the gene tree, an excess of nonsynonymous substitutions was found along the *Hun* branch (Figure 4).

Limiting ourselves to the D. simulans data, the results of these analyses could be interpreted in two ways. One interpretation is that following the duplication, the accumulation of mutations was the result of relaxed functional constraint that was then followed by purifying selection. The second interpretation, and the one that we argue for, is that positive selection for a novel function drove the fixation of nonsynonymous substitutions and that the gene is currently under functional constraint. Evidence for this comes from the fact that despite the considerable number of substitutions that have occurred across this gene, all have maintained an open reading frame, and polymorphisms show a signature of purifying selection that favors synonymous mutations. In addition, our analysis of gene conversion suggested that conversion event(s) have occurred between *Bällchen* and *Hun*. However, most of the shared polymorphism that exists from the conversion event(s) are at synonymous sites (5/7), suggesting that selection may have acted on replacement sites to overcome conversion and to drive the divergence of the paralogs.

### Sex-Specific Expression

Evidence of a new function for *Hun* is also supported by its derived testes-specific expression (Figure 3C–E). How exactly such a drastic change in its expression profile evolved remains a mystery. Further experiments involving the identified UTR regions as well as the putative promoter region may help shed light on this issue. Curiously, *Bällchen* encodes a kinase that is involved in germ cell development, and *D. melanogaster Bällchen* knockout mutants have been observed to cause reduced testes size and recessive male sterility (FlyBase; http://flybase.bio.indiana.edu/). This leads to the tempting but unanswered question of whether or not such data indicate a possible predisposition for testes function for *Hun*. We did ask if there was evidence for the immediate neighbors of *Hun (CG32614* and *CG12454)* having similar expression patterns by querying the GenBank D. simulans and D. melanogaster EST databases [38]. The results of this query were rather uninformative and returned only a single significant match: *CG32614* was represented in adult D. melanogaster head tissue (clone ID RH49655).

A surprising result that has come out of the research on the evolution of new genes is the disproportionate number of new genes that have evolved testes and testes-specific expression [26–29,53]. Many of these are retrogenes that are on autosomes but originated from X-linked parental genes. For example, in a study of 24 functional retrogenes in *Drosophila,* Betrán et al. [26] found nine to have evolved testes-specific expression with an additional five expressed in the testes and other tissues. In a similar study of 45 mammalian retrogenes, Emerson et al. [28] found 16 to have evolved testes-specific expression and an additional seven expressed in testes and other tissues. And again, Marques et al. [29] recently reported that in their dataset of seven primate-specific functional retrogenes all are testes-expressed and one is testis-specific. Because many of the genes in the above studies are involved in sperm-related functions, it is likely that selection is responsible for the pattern.

In contrast to the retrogene movement from the X chromosome to autosomes [26,28], *Hun* originated from a 3R-linked parental gene and duplicated to the X chromosome, where it also evolved testis-specific expression. It is notable that *Hun*'s evolutionary past aligns with the predictions of a sexual antagonism hypothesis as presented by Rice [30]: positive selection, male-specific expression, the duplicate fixation on the X chromosome, and the absence of expression of the gene in the homogametic sex. Sexual antagonism is defined as the situation in which a trait confers an advantage in one sex while conferring a disadvantage to the other. It has been recognized theoretically that sex chromosomes may provide efficient environments under certain selective models (e.g., hemizygous exposure) [52,53]. If a sexually antagonistic gene is favored in the heterogametic sex, X-linkage may increase the probability of increasing in frequency, when rare if the mutation is recessive [30]. The further fixation requires the silencing of the gene in the homogametic sex, as we observed in this case. Our data are consistent with *Hun* being a sexually antagonistic gene and merits further investigation.

### Summary on the Functionality of *Hun*

The criteria for defining pseudogenes can be tricky and is often inconsistent [11,49]. Our interpretation of the data regarding the functionality of *Hun* is that while there is statistical and expression-based evidence that *Hun* is functional in all three species, numerous premature stop codons present in the D. sechellia and D. mauritiana copies evoke concern. Our conservative hypothesis is that *Hun* has evolved new function through positive selection and is constrained in *D. simulans,* but is likely to be in the process of being pseudogenized in these latter two species. We also consider the mutations found in D. sechellia and *D. mauritiana* as additional support for *Hun*'s functionality in D. simulans.

## Materials and Methods

### Identification and verification of gene duplication.

In an initial genome-wide effort to identify new genes in the D. melanogaster subgroup, FISH screens of polytene chromosomes of eight representative species *(Drosophila erecta, D. yakuba, Drosophila santomea, Drosophila teiseri, D. sechellia, D. mauritiana, D. simulans, D. melanogaster)* were carried out using D. melanogaster cDNA probes from *Drosophila* Gene Collection Release 1.0 [31,32]. Probes that produced extra hybridization signals in new cytological sites were considered candidates for new genes and were subjected to further analysis.

### Southern hybridization.

To verify the FISH results, gDNA from the eight species of the D. melanogaster subgroup was extracted and digested with Xho I and BamH I restriction enzymes and then hybridized with the cDNA probes of candidate new genes. The Southern hybridization patterns were compared to the FISH signals and support was found for cases in which the gene copy number agreed in both.

### Database homology search.

tBLASTN searches were carried out against all available D. melanogaster subgroup genomes (the annotated D. melanogaster genome [Flybase release 3], the unassembled D. simulans contigs of strain w501 (downloaded March 8, 2005), the consensus D. simulans syntenic assembly (downloaded March 8, 2005), and the assembled D. yakuba genome (DPGP early user access, downloaded September 29, 2004). tBLASTN searches were done on our local server with standard parameters. The search results potentially provide the chromosomal locations of the duplicates that were identified by the FISH analyses and also provide gDNA sequences with which to design gene-specific primers.

### Genomic DNA amplification and sequencing.

PCR and sequencing were used as the final verification of the duplication of *Bällchen* in *D. simulans, D. sechellia,* and D. mauritiana. Both parental and duplicate-specific PCR and sequencing primers were designed. The entire coding region, as well as some UTRs of both the parental copy and the duplicated copy, was sequenced. All primer sequences are available upon request.

### Analysis of gene expression.

To investigate the expression patterns of *Bällchen* in males and females throughout the D. melanogaster subgroup, and of the duplicate copy within the D. simulans complex, total RNA was extracted from eight strains: Oregon R *(D. melanogaster),* Florida *(D. simulans),* Coyne line *(D. sechellia),* W148g122 *(D. mauritiana),* BRZ8 *(D. tessieri),* Wu line 115 *(D. yakuba),* STO CAGO 1462–12 *(D. santomea),* and Lemeunier line 154.1 *(D. erecta).* 20–30 2- to 4-d-old whole-body adult males and females from each species were used for total RNA extraction (RNeasy mini kit, Qiagen, Valencia, California, United States). Total RNA was treated by RNase-free DNase I (Invitrogen, Carlsbad, California, United States) and reverse-transcribed using Superscript III reverse transcriptase (Invitrogen). cDNA was amplified using gene-specific primers and visualized on 1% agarose gels.

To investigate the tissue-specific expression patterns of *Hun* in *D. simulans, D. sechellia,* and *D. mauritiana,* dissections of 2- to 5-d-old adults were carried out in saline solution. Head, thorax, abdomen, as well as tissue samples from testes and accessory glands plus ejaculatory ducts, were prepared from ~ 40 individuals per sex for D. simulans. For D. sechellia and *D. mauritiana,* only testes and gonadectomized males (males with testes and accessory glands removed) were obtained. Tissue was immediately placed in RNA-later solution (Ambion, Austin, Texas, United States) and put on ice. Total RNA was extracted from each tissue sample and RT-PCR was carried out as described above.

### Amplification of cDNA ends (RACE).

To verify that cDNA is transcribed from the correct strand and to determine the 5′ and 3′ UTR of *Hun,* 5′ RLM-RACE and 3′ RACE (Ambion) were carried out using total RNA from adult males in *D. simulans, D. sechellia,* and D. mauritiana. The cDNA ends were sequenced.

### Sequence analysis: Population genetics and molecular evolution.

To obtain polymorphism data for *Bällchen,* the entire coding region and the only intron were sequenced from ten D. simulans isofemale lines from a single Madagascar population [54]. *Bällchen* was also sequenced from a single *D. melanogaster, D. sechellia,* and D. mauritiana individual for divergence comparisons.

*Hun* reads, which included the start codon and extended to the polyadenylation site, were obtained. Templates for sequencing were amplified from gDNA extractions using a single male adult from each line. Sequence reads from both the forward and reverse strands were obtained, except for the region around an intronic polyT in the *Hun* copy where we had technical difficulties sequencing through from either side. For this ~ 70-bp region, we sequenced in one direction multiple times to obtain at least 2X coverage and no ambiguous sites. In addition, to prove that the sequencing difficulties at the polyT region were not caused by heterozygosity, we cloned and sequenced one line (MD235) using the TOPO cloning kit (Invitrogen). All samples were sequenced using Applied Biosystems 3730XL and 3100 (Foster City, California, United States) automated DNA sequencers. Contig sequences for each line were assembled using CodonCode Aligner (CodonCode Corporation, Dedham, Massachusetts, United States).

Madagascar is believed to be the speciation center for D. simulans [55]. Therefore, in an effort to maximize diversity, we used ten isofemale lines collected from the island for population genetics analysis. Genomic sequences were aligned using ClustalW with default settings [56]. Alignments involving the *D. sechellia Bällchen* copy were generated using ClustalW as well, but with parameters allowing for larger gaps. The DnaSP package [57] was used to estimate DNA sequence variation, as well as for calculating Fu and Li's D [39], Fu and Li's F [39], and Tajima's D [40] and codon bias. We also conducted a test of neutrality by comparing the synonymous and nonsynonymous variation within and between *Bällchen* and *Hun* copies in a McDonald-Kreitman framework [41], using the MK test program [58] and DnaSP [57]. For the McDonald-Kreitman tests, our polymorphism data came from both *Hun* alleles and from pooled homologous regions between *D. simulans Hun* and *Bällchen* alleles.

To investigate the selective forces acting on *Bällchen* and *Hun* on the molecular evolution scale, we estimated the statistic Ka/Ks, where Ka is the number of nonsynonymous substations per nonsynonymous site and Ks is the number of synonymous substations per synonymous site, for each paralog pair within D. simulans using Codeml in PAML [59]. Ka/Ks values significantly greater than one are often taken as evidence for positive selection, while values significantly less than one are often taken as evidence of constraint. We also tested for functional constraint on *Hun* using our polymorphism data. To do this we calculated the proportion of nonsynonymous and synonymous sites found in our *Hun* population dataset. In order to obtain a neutral expectation to test against, we multiplied the total observed polymorphisms from the same dataset by these two proportions. Under neutrality these mutations are expected to be distributed randomly. A chi-square test was then carried out between the observed and expected values [11].

### Promoter and signal peptide prediction.

To determine if any identifiable promoter region is present near the *Hun* locus, NNPP 2.2 [37] was used with default settings to analyze > 1 kb of *Hun* 5′ flanking sequence. All D. simulans protein sequences were checked for evidence of a signal peptide using the signalP 3.0 server [60].

### Direct repeat and polyA search.

It may be possible to observe a signature of the mechanism for gene duplication. For example, retrogenes lack the parental gene's introns and often posses a 3′ polyA tract, while duplicates arising through a transposon intermediate will be flanking by direct repeats. To search for a polyA tract, we manually inspected sequence surrounding the novel 3′ junction. To search for direct repeats, REPuter was used [36] to analyze ~ 1.4 kb of 3′ and 5′ sequence. REPuter is capable of searching for imperfect repeat sequences by allowing for mismatches, insertions, and deletions.

### Tests for gene conversion.

While divergence of paralogs may occur following duplication, an alternative possibility is that gene conversion will homogenize the pair and lead to the concerted evolution of genes in the family [43,61,62]. Because selection and conversion are opposing forces, it is thought that selection may need to be strong to overcome conversion [43,61]. Data that are often used to infer conversion events are the presence of conversion tracts and the presence of shared polymorphism. To detect gene conversion between *Hun* and *Bällchen* we used both. Variation was placed into one of three categories: private polymorphism, where polymorphism falls in only one or the other gene; fixed divergence, where each gene is fixed for different nucleotides; and shared polymorphism, where nucleotides are segregating in both genes [61,63]. We calculated the number for each category, on both nucleotide and amino acid levels, from alignments of the homologous region between *Bällchen* and *Hun* using the sharedPoly program [58]. For this analysis all sites containing more than two states were removed. We also estimated the population rate of ectopic gene conversion, *Ĉ* (3.5N_e_c), on the same dataset using the estimators of Innan [43], code kindly provided by K. Thornton. *Ĉ* is slightly lower than 3.5 N_e_c for this autosome-X chromosome scenario according to a simulation by H. Innan and S. Takuro (personal communication). Finally, to detect conversion tracts, we used the GENECONV program [44,64] with default settings on the same dataset. GENECONV conducts pair-wise analyses and does not utilize the population data.

## Supporting Information

Figure S1 Alignment of *Bällchen*'s and *Hun*'s Coding Regions plus Additional Flanking Sequence Alignment displays the homologous duplicated region and the newly acquired X chromosome sequence. Yellow bars indicate acquired UTR sequence, light blue bars indicate acquired protein-coding region. Italicized pink sequence is 5′ -most regions ascertained by RACE, italicized red sequence is the shared start codon, purple box surrounds 3′ end of homology, italicized black sequences are stop codons, italicized light green sequence is *Hun*'s polyadenylation site, and italicized blue sequence is a putative 5′ nick site.
(1.1 MB JPG)

Figure S2 Alignment of *Hun*'s 5′ UTR Region as Characterized by RLM-RACE Product Aligned to gDNA Sequence The region between arrows indicates the acquired intron, and the pink sequence marks start codons. The red bar underlines the predicted promoter region with the red highlighted “A” noting the predicted transcriptional start site. This promoter prediction is consistent with our 5′ RLM-RACE experiments within a single base.
(456 KB JPG)

Figure S3 Alignment of *Hun* from *D. mauritiana, D. sechellia,* and D. simulans
Alignment displays the large deletions that have occurred along the D. sechellia branch and its seven premature stop codons, underlined in red. The single base deletion in D. mauritiana is circled in purple and its six premature stop codons are underlined in green. The shared stop codon is marked by the black box.
(989 KB JPG)

Figure S4 Polymorphism Table for *Hun*
(302 KB JPG)

Figure S5 Polymorphism Table for *Bällchen*
(322 KB JPG)

Table S1 Ka/Ks Values between *Hun* and *Bällchen* within Each Strain (255 KB JPG)

### Accession Numbers

 All sequences have been deposited in the GenBank database and have been assigned the accession numbers DQ438912–DQ438935.

## Abbreviations

bp: base pair
FISH: fluorescent in situ hybridization
gDNA: genomic DNA

## References

[pgen-0020077-b001] Fisher RA (1935). The sheltering of lethals. Am Nat.

[pgen-0020077-b002] Haldane JBS (1933). The part played by recurrent mutation in evolution. Am Nat.

[pgen-0020077-b003] Muller JJ (1936). Bar duplication. Science.

[pgen-0020077-b004] Ohno S (1970). Evolution by gene duplication.

[pgen-0020077-b005] Gilbert W (1978). Why genes in pieces?. Nature.

[pgen-0020077-b006] Patthy L (1999). Genome evolution and the evolution of exon shuffling: A review. Gene.

[pgen-0020077-b007] Jones C, Custer AW, Begun DJ (2005). Origin and evolution of a chimeric fusion gene in *Drosophila subobscura, D. madeirensis,* and D. guanche. Genetics.

[pgen-0020077-b008] Kaessmann H, Zollner S, Nekrutenko A, Li W (2002). Signatures of domain shuffling in the human genome. Genome Res.

[pgen-0020077-b009] Rajalingam R, Parham P, Abi-Rached L (2004). Domain shuffling has been the main mechanism forming new hominoid killer cell Ig-like receptors. J Immunol.

[pgen-0020077-b010] Patthy L (1995). Protein evolution by exon shuffling.

[pgen-0020077-b011] Long MY, Langley CH (1993). Natural selection and the origin of jingwei, a chimeric processed functional gene in *Drosophila*. Science.

[pgen-0020077-b012] Li WH (1997). Molecular evolution.

[pgen-0020077-b013] Loppin B, Lepetit D, Dorus S, Couble P, Karr TL (2005). Origin and neofunctionalization of a *Drosophila* paternal effect gene essential for zygote viability.

[pgen-0020077-b014] Long M, Betran E, Thornton K, Wang W (2003). The origin of new genes: Glimpses from the young and old. Nat Rev Genet.

[pgen-0020077-b015] Allgood ND, Silhavy TJ, Kucherlapati R, Simth GR (1988). Illegitimate recombination in bacteria. Genetic recombination.

[pgen-0020077-b016] Roth D, Wilson J, Kucherlapati R, Simth GR (1988). Illegitimate recombination in mammalian cells. Genetic recombination.

[pgen-0020077-b017] Roth DB, Porter TN, Wilson JH (1985). Mechanisms of nonhomologous recombination in mammalian cells. Mol Cell Biol.

[pgen-0020077-b018] Roth DB, Wilson JH (1986). Nonhomologous recombination in mammalian cells: Role for short sequence homologies in the joining reaction. Mol Cell Biol.

[pgen-0020077-b019] van Rijk A, Bloemendal H (2003). Molecular mechanisms of exon shuffling: Illegitimate recombination. Genetica.

[pgen-0020077-b020] van Rijk A, de Jong WW, Bloemendal H (1999). Exon shuffling mimicked in cell culture. Proc Nat Acad Sci U S A.

[pgen-0020077-b021] Kumatori A, Faizunnessa NN, Suzuki S (1998). Nonhomologous recombination between the cytochrome b(558) heavy chain gene (CYBB) and LINE-1 causes an X-linked chronic granulomatous disease. Genomics.

[pgen-0020077-b022] Hu X, Worton RG (1992). Partial gene duplication as a cause of human disease. Hum Mutat.

[pgen-0020077-b023] Zucman-Rossi J, Legoix P, Victor JM, Lopez B, Thomas G (1998). Chromosome translocations based on illegitimate recombination in human tumors. Proc Nat Acad Sci U S A.

[pgen-0020077-b024] Zhang J, Dean AM, Brunet F, Long M (2004). Evolving protein functional diversity in new genes of *Drosophila*. Proc Nat Acad Sci U S A.

[pgen-0020077-b025] Nurminsky DI, Nurminskaya MV, De Aguiar D, Hartl DL (1998). Selective sweep of a newly evolved sperm-specific gene in *Drosophila*. Nature.

[pgen-0020077-b026] Betran E, Thornton K, Long M (2002). Retroposed new genes out of the X in *Drosophila*. Genome Res.

[pgen-0020077-b027] Betran E, Emerson JJ, Kaessmann H, Long M (2004). Sex chromosomes and male functions: Where do new genes go?. Cell Cycle.

[pgen-0020077-b028] Emerson JJ, Kaessmann H, Betran E, Long M (2004). Extensive gene traffic on the mammalian X chromosome. Science.

[pgen-0020077-b029] Marques AC, Dupanloup I, Vinckenbosch N, Reymond A, Kaessmann H (2005). Emergence of young human genes after a burst of retroposition in primates. PLoS Biol.

[pgen-0020077-b030] Rice WR (1984). Sex chromosomes and the evolution of sexual dimorphism. Evolution.

[pgen-0020077-b031] Wang W, Brunet FG, Nevo E, Long M (2002). Origin of sphinx, a young chimeric RNA gene in Drosophila melanogaster. Proc Nat Acad Sci U S A.

[pgen-0020077-b032] Wang W, Yu HJ, Long MY (2004). Duplication-degeneration as a mechanism of gene fission and the origin of new genes in *Drosophila* species. Nat Genet.

[pgen-0020077-b033] Brudno M, Do CB, Cooper GM, Kim MF, Davydov E (2003). LAGAN and Multi-LAGAN: Efficient tools for large-scale multiple alignment of genomic DNA. Genome Res.

[pgen-0020077-b034] Brudno M, Malde S, Poliakov A, Do C, Courone O (2003). Glocal alignment: Finding rearrangements during alignment. Special Issue on the Proceedings of the ISMB, Bioinformatics.

[pgen-0020077-b035] Couronne O, Poliakov A, Bray N, Ishkhanov T, Ryaboy D (2002). Strategies and tools for whole-genome alignments. Genome Res.

[pgen-0020077-b036] Kurtz S, Choudhuri JV, Ohlebusch E, Schleiermacher C, Stoye J (2001). REPuter: The manifold applications of repeat analysis on a genomic scale. Nucleic Acids Res.

[pgen-0020077-b037] Reese MG (2001). Application of time-delay neural network to promoter annotation in the Drosophila mealanogaster genome. Comput Chem.

[pgen-0020077-b038] Boguski M, Lowe TM, Tolstoshev CM (1993). dbEST-database for expressed sequence tags. Nat Genet.

[pgen-0020077-b039] Fu YX, Li WH (1993). Statistical tests of neutrality of mutations. Genetics.

[pgen-0020077-b040] Tajima F (1989). Statistical method for testing the neutral mutation hypothesis by DNA polymorphism. Genetics.

[pgen-0020077-b041] McDonald JH, Kreitman M (1991). Adaptive protein evolution at the Adh locus in *Drosophila*. Nature.

[pgen-0020077-b042] Birney E (2004). Available: ftp://ftp.ebi.ac.uk/pub/software/unix/wise2. Accessed 21 April 2006.

[pgen-0020077-b043] Innan H (2003). The coalescent and infinite-site model of a small multigene family. Genetics.

[pgen-0020077-b044] Sawyer S (1999). GENECONV: A computer package for the statistical detection of gene conversion.

[pgen-0020077-b045] Katju V, Lynch M (2003). The structure and early evolution of recently arisen gene duplicates in the Caenorhabditis elegans genome. Genetics.

[pgen-0020077-b046] Borgato L, Bonizzato A, Lunardi C, Dusi S, Andrioli G (2001). A 1.1-kb duplication in the p67-phox gene causes chronic granulomatous disease. Hum Genet.

[pgen-0020077-b047] Zhu J, Schiestl RH (1996). Topoisomerase I involvement in illegitimate recombination in Saccharomyces cerevisiae. Mol Cell Biol.

[pgen-0020077-b048] Richardson C, Moynahan ME (1998). Double-strand break repair by interchromosomal recombination: suppression of chromosomal translocations. Genes Dev.

[pgen-0020077-b049] Begun DJ (1997). Origin and evolution of a new gene descended from alcohol dehydrogenase in Drosophila. Genetics.

[pgen-0020077-b050] Liu Q, Brubaker CL, Green AG, Marshall DR, Sharp PJ (2001). Evolution of the FAD2–1 fatty acid desaturase 5′ UTR intron and the molecular systematics of Gossypium (Malvaceae). Am J Bot.

[pgen-0020077-b051] Morello L, Bardini M, Sala F, Breviario D (2002). A long leader intron of the Ostub16 rice beta-tubulin gene is required for high-level gene expression and can autonomously promote transcription both in vivo and in vitro. Plant J.

[pgen-0020077-b052] Charlesworth B, Coyne JA, Barton NH (1987). The relative rates of the evolution of sex chromosomes and autosome. Am Nat.

[pgen-0020077-b053] Gibson J, Chippindale AK, Rice WR (2002). The X chromosome is a hot spot for sexually antagonistic fitness variation. Proc Biol Sci.

[pgen-0020077-b054] Dean MD, Ballard JWO (2004). Linking phylogenetics with population genetics to reconstruct the geographic origin of a species. Mol Phylogenet Evol.

[pgen-0020077-b055] Lachaise D, Cariou ML, David JR, Lemeunier F, Tsacas L (1988). Historical biogeography of the Drosophila melanogaster species subgroup. Evol Biol.

[pgen-0020077-b056] Chenna R, Sugawara H, Koike T, Lopez R, Gibson TJ (2003). Multiple sequence alignment with the Clustal series of programs. Nucleic Acids Res.

[pgen-0020077-b057] Rozas J, Sánchez-DelBarrio JC, Messeguer X, Rozas R (2003). DnaSP, DNA polymorphism analyses by the coalescent and other methods. Bioinformatics.

[pgen-0020077-b058] Thornton K (2003). Libsequence: A C++ class library for evolutionary genetic analysis. Bioinformatics.

[pgen-0020077-b059] Yang ZH (1997). PAML: A program package for phylogenetic analysis by maximum likelihood. Comput Appl Biosci.

[pgen-0020077-b060] Bendtsen J, Nielsen H, von Heijne G, Brunak S (2004). Improved prediction of signal peptides: SignalP 3.0. J Mol Biol.

[pgen-0020077-b061] Innan H (2003). A two-locus gene conversion model with selection and its application to the human RHCE and RHD genes. Proc Nat Acad Sci U S A.

[pgen-0020077-b062] Walsh B (2003). Population-genetic models of the fates of duplicate genes. Genetica.

[pgen-0020077-b063] Thornton K, Long M (2005). Excess of amino acid substitutions relative to polymorphism between X-linked duplications in Drosophila melanogaster. Mol Biol Evol.

[pgen-0020077-b064] Sawyer S (1989). Statistical tests for detecting gene conversion. Mol Biol Evol.

